# Low cardiac index and stroke volume on admission are associated with poor outcome in critically ill burn patients: a retrospective cohort study

**DOI:** 10.1186/s13613-016-0192-y

**Published:** 2016-09-13

**Authors:** Sabri Soussi, Benjamin Deniau, Axelle Ferry, Charlotte Levé, Mourad Benyamina, Véronique Maurel, Maïté Chaussard, Brigitte Le Cam, Alice Blet, Maurice Mimoun, Jêrome Lambert, Marc Chaouat, Alexandre Mebazaa, Matthieu Legrand, for the PRONOBURN group

**Affiliations:** 1 Department of Anesthesiology and Critical Care and Burn Unit, AP-HP, Hôpital Saint-Louis, 1 Avenue Claude Vellefaux, 75010 Paris, France; 2 Plastic Surgery and Burn Unit, AP-HP, Hôpital Saint-Louis, Paris, France; 3 Hôpital Lariboisière, UMR INSERM 942, Institut National de la Santé et de la Recherche Médicale (INSERM), Paris, France; 4 Université Paris Diderot, 75475 Paris, France; 5 Department of Biostatisitcs, AP-HP, Hôpital Saint-Louis, Paris, France

**Keywords:** Burns, Hemodynamics, Monitoring, Outcome, Organ dysfunction, Mortality

## Abstract

**Background:**

Impact of early systemic hemodynamic alterations and fluid resuscitation on outcome in the modern burn care remains controversial. We investigate the association between acute-phase systemic hemodynamics, timing of fluid resuscitation and outcome in critically ill burn patients.

**Methods:**

Retrospective, single-center cohort study was conducted in a university hospital. Forty critically ill burn patients with total body surface area (TBSA) burn-injured >20 % with invasive blood pressure and cardiac output monitoring (transpulmonary thermodilution technique) within 8 h from trauma were included. We retrospectively examined hemodynamic variables during the first 24 h following admission, and their association with 90-day mortality.

**Results:**

The median (interquartile range 25th–75th percentile) TBSA, Simplified Acute Physiology Score II (SAPS II) and Abbreviated Burn Severity Index of the study population were 41 (29–56), 31 (23–50) and 9 (7–11) %, respectively. 90-Day mortality was 42 %. There was no statistical difference between the median pre-hospital and 24-h administered fluid volume in survivors and non-survivors. On admission, stroke volume (SV), cardiac index (CI), oxygen delivery index and mean arterial pressure (MAP) were significantly lower in patients who died despite similar fluid resuscitation volume. ROC curves comparing the ability of initial SV, CI, MAP and lactate to discriminate 90-day mortality gave areas under curves of, respectively, 0.89 (CI 0.77–1), 0.77 (CI 0.58–0.95), 0.73 (CI 0.53–0.93) and 0.78 (CI 0.63–0.92); (*p* value <0.05 for all). In multivariate analysis, SAPS II and initial SV were independently associated with 90-day mortality (best cutoff value for SV was 27 mL, sensitivity 92 %, specificity 69 %). During 24 h, no interaction was found between time and outcome regarding macrocirculatory parameters changes. Hemodynamic parameters improved during the first 24-h resuscitation in all patients but patients who died had lower SV and CI on admission, which remained through the first 24 h.

**Conclusion:**

Low initial SV and CI were associated with poor outcome in critically ill burn patients. Very early hemodynamic monitoring may in help detecting under-resuscitated patients. Future prospective interventional studies should explore the impact of early goal-directed therapy in these specific patients.

**Electronic supplementary material:**

The online version of this article (doi:10.1186/s13613-016-0192-y) contains supplementary material, which is available to authorized users.

## Background

 Fluid resuscitation is considered a cornerstone of initial management of burn patients. Burn patients exhibit initial low intravascular volume, high systemic vascular resistance and low cardiac output making fluid loading the first-line treatment [1, 2]. While early fluid resuscitation has been associated with survival in children with burns [3], volume and timing of fluid resuscitation have been a matter of debate in adult severe burn patients. Although the Parkland formula has long been considered the gold standard, there is now accumulating evidence that fluid requirements estimated by this formula are inaccurate [4]. Goal-directed therapy (GDT) based on more advanced hemodynamic monitoring has been suggested to offer a survival benefit to burn patients [5]. Physiological and clinical studies suggest that both profound hypovolemia and over-resuscitation leading to a positive fluid balance are associated with poor outcomes in critically ill burn patients [3, 6, 7]. However, hemodynamic targets of initial resuscitation in severely ill burn patients remain largely unexplored.

Therefore, in the present cohort study we aimed to investigate the association between acute-phase systemic hemodynamics, timing of fluid resuscitation and outcome in critically ill burn patients. We hypothesized that inadequate initial (within 8 h from trauma) intravascular volume and under-resuscitation may lead to multiple organ dysfunction and mortality in critically ill burn patients.

## Methods

### Study design and eligibility

We conducted a retrospective, single-center cohort study in the Burn Unit of the Saint Louis Hospital, Paris, France. The study was approved by our local ethical committee (comité de protection des personnes IV, St-Louis Hospital; Institutional Review Board 00003835, protocol 2013/17NICB). All medical records of the patients admitted to our intensive care burn unit (ICBU) between March 2013 and October 2014 were screened. Burn patients meeting all the following criteria were included in the study: total body surface area burn (TBSA burn) >20 %, delay from the time of thermal injury to the time of ICBU <8 h, patients with invasive blood pressure and continuous cardiac output monitoring within the first hour after admission, patients with a central venous catheter (CVC) in the superior vena cava (SVC) territory. Exclusion criteria were: patients with chemical or electrical burns, coexisting non-burn trauma, patients moribund on admission or dead within 72 h from admission, and patients with do-not-resuscitate orders.

### Study endpoints

The primary endpoint for the present study was 90-day mortality. The secondary endpoint was the onset of early multiple organ dysfunction syndrome (MODS) defined a priori as a Sequential Organ Failure Assessment (SOFA) score >8 at any point up to day 4 after admission [8, 9].

### Data collection

The authors collected the following data: age, sex, body mass index (BMI), TBSA, full-thickness BSA burned, mechanism of injury and patients’ characteristics, Simplified Acute Physiology Score II (SAPS II), Abbreviated Burn Severity Index (ABSI) [10], different treatments in the first 7 days after admission, diagnosis of sepsis, 28 and 90 day mortality, acute kidney injury (AKI) within the first 7 days evaluated by the kidney disease improving global outcomes (KDIGO) criteria [11] and daily organ dysfunction evaluated by using the SOFA score within the first 7 days after admission [12]. Early MODS was defined a priori as a SOFA score >8 at any point up to day 4 after admission [9, 13].

The following admission hemodynamic, oxygenation and metabolic variables were examined: heart rate (HR), cardiac index (CI), stroke volume (SV), invasive systolic arterial pressure (SAP), invasive mean arterial pressure (MAP), invasive diastolic arterial pressure (DAP), systemic vascular resistance index (SVRI), central venous pressure (CVP), urine output (UO), central venous oxygen saturation (ScvO_2_), oxygen delivery index (DO_2_I), central venous–arterial PCO_2_ difference (PCO_2_ gap) arterial serum lactate level and its clearance. Lactate clearance (%) was defined using the following formula: lactate on admission (hour 0) − lactate at hour 6 (H6), divided by lactate on admission, then multiplied by 100. A positive value denotes a decrease or clearance of lactate, whereas a negative value denotes an increase in lactate 6 h after admission: lactate clearance (%) = (lactate H0 − lactate H6) × 100/lactate H0 [14]. Central PCO_2_ gap was calculated as the difference between central venous and arterial partial pressure of carbon dioxide, respectively, obtained at the same time from CVC and arterial blood samples. Otherwise, DO_2_I was calculated by the application of the standard formulae and indexed to body surface area.

### Patient management

Hemodynamic targets within 24 h of admission were defined by the Saint Louis Hospital ICBU resuscitation protocol: MAP >65 mmHg, 0.5 mL/kg/h < UO < 1 mL/kg/h, 2.5 L/min/m^2^ < CI < 3 L/min/m^2^ and ScvO_2_ >70 %. Norepinephrine was administered when required (DAP <50 mmHg and/or SVRI <1250 dynes s/cm^5^/m^2^). Patients received initial fluid resuscitation using intravenous Ringer’s Lactate of 0.25 mL/kg/%TBSA/h (which corresponds to the 2 mL/kg/%TBSA in the first 8 h of the Parkland formula) with additional fluid loading to reach predefined hemodynamic targets. Cardiac function was systematically assessed on admission by echocardiography. All these variables were monitored continuously and checked hourly except for the ScvO_2_ which was measured at least every 4–6 h on blood samples from the CVC using a blood gas analyzer (Cobas b 123 POC system, Roche Diagnostics, USA). CI was measured by transpulmonary thermodilution (TPTD) with a PiCCO monitor (PiCCO-2 Pulsion Medical Systems AG, Munich, Germany). The PiCCO monitor was calibrated every 2 h in the acute-phase patients during the first 48 h.

Twenty percent albumin was administrated in patients with TBSA >30 % after the 6th h after thermal injury to reach a serum albumin concentration of 25–30 g/l. When mechanical ventilation was initiated, tidal volume was limited to 6–7 mL/kg while maintaining an inspiratory plateau pressure <30 cmH_2_O. Early enteral nutrition was initiated within 24 h. Glycemic control was adjusted to maintain glucose levels between 5 and 9 mmol/L. Surgical treatment included escharotomy or fasciotomy as needed and early coverage of excised burn wounds with autografts and/or allografts within the first 7 days after admission as the clinical condition of the patient permitted.

### Statistical analysis

Quantitative parameters are reported as median and interquartile range (IQR 25th–75th percentile), and qualitative parameters are expressed as number and percentage. Categorical variables were compared using the Chi-square test or Fisher’s exact test as appropriate. Continuous variables were compared using the Mann–Whitney *U* test. For 90-day mortality discrimination, the receiver operating characteristic curve (ROC) analysis tested the best threshold values (using the Youden index) of the variables and the area under the curve (AUC) was calculated. The significance of the difference between the AUCs was assessed using the De Long and De Long test. Differences at a level of *p* < 0.05 were considered statistically significant. Variables associated with 90-day survival in univariate analysis were entered in a multivariate logistic regression model to identify factors independently associated with the outcome. Considering the rule of a minimum of 5–10 events for each predictor variable considered in the model [15], when several related variables were associated with the outcome in univariate analysis, only the most clinically relevant were included in the multivariate model. Repeated-measures analysis of variance (repeated-measures ANOVA) was used to determine whether hemodynamic and oxygenation variables changed with time. To test whether patients’ hemodynamic profiles differed between patients alive and dead at 90 days, linear mixed effect models were fitted with time of measurement and survival status as fixed effect and random intercept and random slope to account for patient-specific effect. A fixed interaction term between survival status and time was added to look for differences within the first hours between patients alive and dead at day 90. The analyses were performed using the SPSS 17.0 software (SPSS, Chicago, IL, USA).

## Results

### Patients’ characteristics

Forty patients fulfilled the inclusion criteria and were included in the study. The screening flowchart of the study population is represented in the Additional file 1: Figure S1. The study patients’ characteristics are summarized in Table 1.

**Table 1:** Patients’ characteristics

Characteristics	All patients (*n* = 40)	Survivors day 90 (*n* = 23)	Non-survivors day 90 (*n* = 17)	*p*
Age (years)	48 (34–58)	45 (30–60)	53 (37–69)	0.004
Male, *n* (%)	26 (65 %)	15 (68 %)	11 (61 %)	0.64
BMI (kg/m^2^)	28 (25–32)	28 (25–31)	30 (24–36)	0.51
Comorbidities, *n* (%)				
Hypertension	8 (20 %)	2 (9 %)	6 (33 %)	0.07
Heart failure	1 (2.5 %)	0 (0 %)	1 (6 %)	0.26
Diabetes mellitus	2 (5 %)	0 (0 %)	2 (12 %)	0.11
COPD	2 (5 %)	1 (4.5 %)	1 (6 %)	0.88
CKD	2 (5 %)	1 (4.5 %)	1 (6 %)	0.26
Stroke	3 (7.5 %)	1 (4.5 %)	2 (12 %)	0.43
TBSA (%)	41 (29–56)	36 (30–42)	59 (42–76)	0.013
Full-thickness BSA burned (%)	21 (10–14)	20 (10–30)	49 (33–65)	0.016
Inhalation injury, *n* (%)	16 (40 %)	6 (27 %)	10 (55 %)	0.07
Delay from trauma (h)	3 (2–5)	3 (1–5)	2 (1–3)	0.45
SAPS II	31 (23–50)	26 (10–42)	50 (31–69)	0.004
ABSI	9 (7–11)	8 (7–9)	12 (10–14)	0.008
SOFA score on day 1	3 (1–4)	1 (0–2)	4 (2–6)	0.01
ICU length of stay (days)	34 (18–53)	42 (20–64)	22 (8–36)	0.01
Mechanical ventilation, *n* (%)	34 (90 %)	18 (78 %)	16 (94 %)	0.4
Use of norepinephrine in the first 24 h, *n* (%)	11 (29 %)	5 (22 %)	6 (35 %)	0.44
Sepsis in the first 7 days, *n* (%)	22 (55 %)	11 (47 %)	11 (64 %)	0.56
Surgery in the first 24 h, *n* (%)^a^	17 (42.5 %)	7 (30 %)	10 (59 %)	0.13
Pre-hospital administered fluid volume (mL/kg/h/%TBSA)	0.15 (0.10–0.23)	0.16 (0.13–0.23)	0.14 (0.06–0.30)	0.48
Administered fluid volume in the first 24 h (mL/kg/%TBSA)	3.7 (2.2–4.8)	3.5 (1.8–4.7)	3.8 (2.6–6.0)	0.57

Data are median (interquartile range 25th–75th percentile), or *n* (%)

*BMI* body mass index, *COPD* chronic obstructive pulmonary disease, *CKD* chronic kidney disease, *TBSA* total body surface area burn, *BSA* body surface area, *SAPS II* Simplified Acute Physiology Score II, *ABSI* Abbreviated Burn Severity Index, *SOFA* Sequential Organ Failure Assessment, *ICU* intensive care unit

^a^Escharotomy and/or fasciotomy

### Association between initial hemodynamic and oxygenation parameters and outcome

In the study population, the 90-day mortality was 42 %. In all cases, death was due to sepsis-related multiple organ failure. The survival curve from admission to day 90 is represented in the Additional file 2: Figure S2. The tested hemodynamic and oxygenation parameters on admission of survivors and non-survivors are compared in Table 2.

**Table 2:** Hemodynamic, oxygenation and metabolic variables on admission

Hemodynamic parameters	All patients (*n* = 40)	Survivors day 90 (*n* = 23)	Non-survivors day 90 (*n* = 17)	*p*
SAP (mmHg)	114 (98–128)	121 (106–156)	114 (97–125)	0.15
DAP (mmHg)	72 (57–81)	76 (62–93)	72 (50–83)	0.06
MAP (mmHg)	80 (63–93)	85 (75–116)	75 (60–93)	0.05
CVP (mmHg)	9 (7–12)	10 (9–11)	9 (7–11)	0.92
UO (mL/kg/h)	0.6 (0.3–1.8)	0.9 (0.3–2.4)	0.4 (0.2–1.8)	0.68
HR (beats/min)	98 (87–112)	94 (87–105)	112 (73–126)	0.91
CI (L/min/m^2^)	2.5 (1.9–3.0)	2.9 (2.5–3.4)	2.0 (1.5–2.4)	0.04
SV (mL/beat)	26 (22–35)	37 (27–43)	22 (20–25)	0.001
SVRI (dynes s/cm^5^/m^2^)	2414 (1932–2881)	2385 (1685–3346)	2548 (2264–3918)	0.97
ScvO_2_ (%)	80 (74–87)	74 (65–76)	84 (78–90)	0.61
DO2I (mL/min/m^2^)	485 (302–580)	537 (381–686)	437 (247–487)	0.001
PCO_2_ gap (mmHg)	7 (4–11)	7 (5–11)	7 (4–11)	0.51
Base deficit (mmol/L)	−3.7 (−7.7 to −0.4)	−3.5 (−5 to 0.5)	−4.5 (−8 to 2)	0.18
Lactate (mmol/L)	3.8 (1.5–5.3)	3.1 (1.4–3.8)	4 (2.6–4.9)	0.025
Number of hemodynamic targets reached at the 6th h after admission, *n* (%)^a^				0.007
1	13 (32.5 %)	3 (13 %)	10 (59 %)	
2	12 (30 %)	7 (30 %)	5 (29 %)	
3	15 (37.5 %)	12 (52 %)	3 (17 %)	

Data are median (interquartile range 25th–75th percentile), or *n* (%)

*SAP* systolic arterial pressure, *DAP* diastolic arterial pressure, *MAP* mean arterial pressure, *CVP* central venous pressure, *UO* urine output, *HR* heart rate, *CI* cardiac index, *SV* stroke volume, *SVRI* systemic vascular resistance index, *ScvO*_*2*_ central venous oxygen saturation, *DO*_*2*_ *I* oxygen delivery index, *PCO*_*2*_ *gap* central venous–arterial PCO_2_ difference

^a^Hemodynamic targets: MAP ≥65 mmHg, UO ≥0.5 mL/kg/h and CI ≥2.5 L/min/m^2^

Initial in-hospital SV, CI and MAP were significantly lower in patients who died [respectively, 22 (20–25) vs 37 (27–43) mL; (*p* = 0.001), 2.0 (1.5–2.4) vs 2.9 (2.5–3.4) L/min/m^2^; (*p* = 0.04) and 75 (60–93) vs 85 (75–116) mmHg; (*p* = 0.05)]. Initial in-hospital DO_2_I was also significantly lower in patients who died [respectively, 437 (247–487) vs 537 (381–686) mL/min/m^2^; (*p* = 0.001)]. All patients had repeated echocardiography during the first 24 h. None of the patients had acute *cor pulmonale* during the first 24 h.

**Fig. 1:**
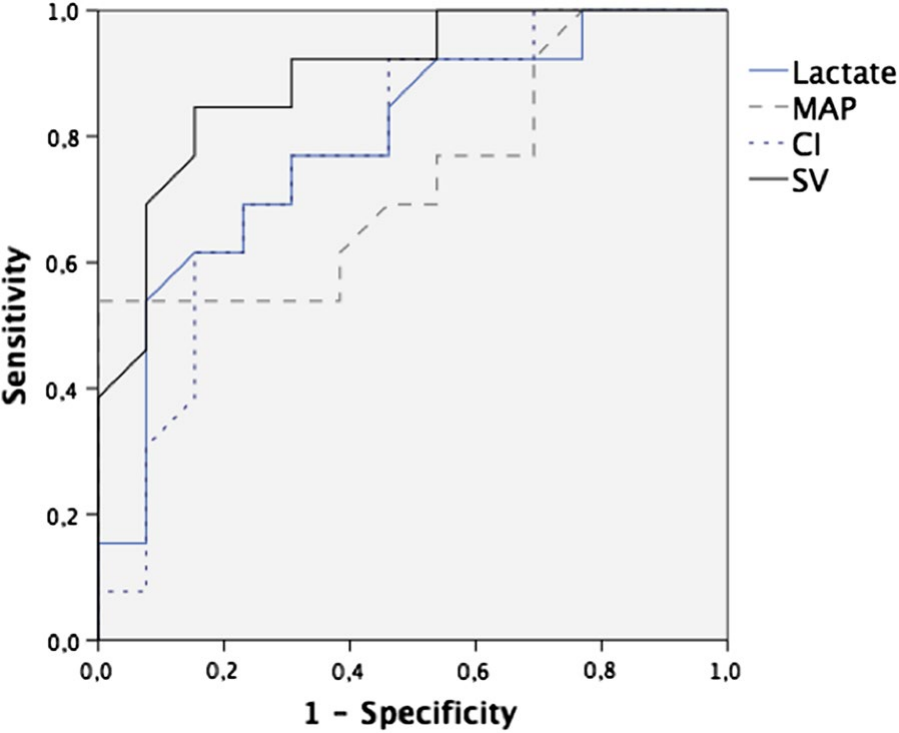
ROC curves comparing the ability of admission SV, CI, MAP and lactate to discriminate 90-day mortality. Areas under the curve for 90-day mortality was, respectively, 0.89 (CI 0.77–1), 0.77 (CI 0.58–0.95), 0.73 (CI 0.53–0.93) and 0.78 (CI 0.63–0.92); (*p* value <0.05 for all) for admission stroke volume, cardiac index, mean arterial pressure and lactate. *SV* stroke volume, *CI* cardiac index, *MAP* mean arterial pressure

Areas under the ROC curves for 90-day mortality were, respectively, 0.89 (CI 0.77–1), 0.77 (CI 0.58–0.95) and 0.73 (CI 0.53–0.93); (*p* value <0.05 for all) for SV, CI and MAP, respectively (Fig. 1). The best cutoff value found for SV was 27 mL (sensitivity 92 %, specificity 69 %). Furthermore, ROC AUCs of SV and CI were not significantly different (*p* value = 0.23).

Using a multiple regression analysis including TBSA, SAPS II and SV, only SAPS II and initial SV were independently associated with 90-day mortality (Table 3).

**Table 3:** Multiple regression analysis to identify factors independently associated with 90-days mortality

Variables and scores	*β*-Coefficient	OR	95 % CI	*p*
On admission SV	−0.36	0.69	0.5; 0.95	0.02
SAPS II	0.16	1.18	1; 1.39	0.04
TBSA (%)	0.01	1	0.95; 1.08	0.64

*OR* odds ratio, *CI* confidence interval, *SV* stroke volume, *SAPS II* Simplified Acute Physiology Score II, *TBSA* total body surface area burn

**Fig. 2:**
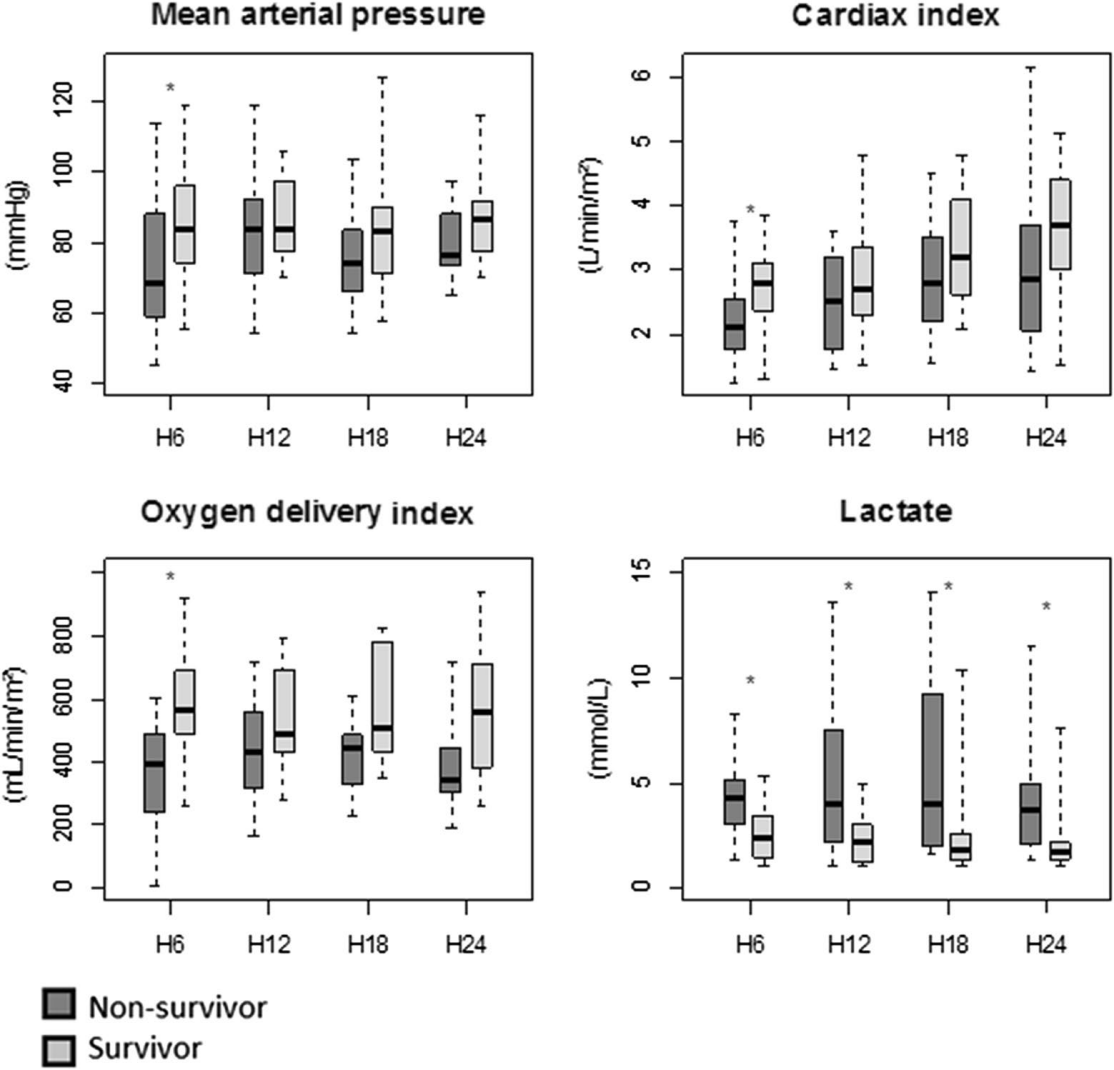
Changes over time of macrocirculatory and oxygenation parameters, and lactate measured over the first 24 h after admission for the survivors and non-survivors (*stars* indicate significant changes over time). Data are shown as median and interquartile range (IQR 25th–75th percentile)

Changes over time of macrocirculatory and oxygenation parameters, and lactate measured over the first 24 h after admission for the survivors and non-survivors, are represented in Fig. 2.

No statistical differences were observed between survivors and non-survivors with regard to initial UO, CVP, BD, ScvO_2_ or PCO_2_ gap.

### Association between initial serum lactate level and outcome

On admission, serum lactate level was significantly higher in patients who died [4.0 (2.6–4.9) vs 3.1 (1.4–3.8) mmol/L; (*p* = 0.025)]. AUC for lactate was 0.78 (CI 0.63–0.92); (*p* = 0.003) to predict 90-day mortality with a cutoff value of 3.8 mmol/L (Fig. 1).

### Changes in macrocirculatory and oxygenation parameters, and lactate during the first 24 h

**Fig. 3:**
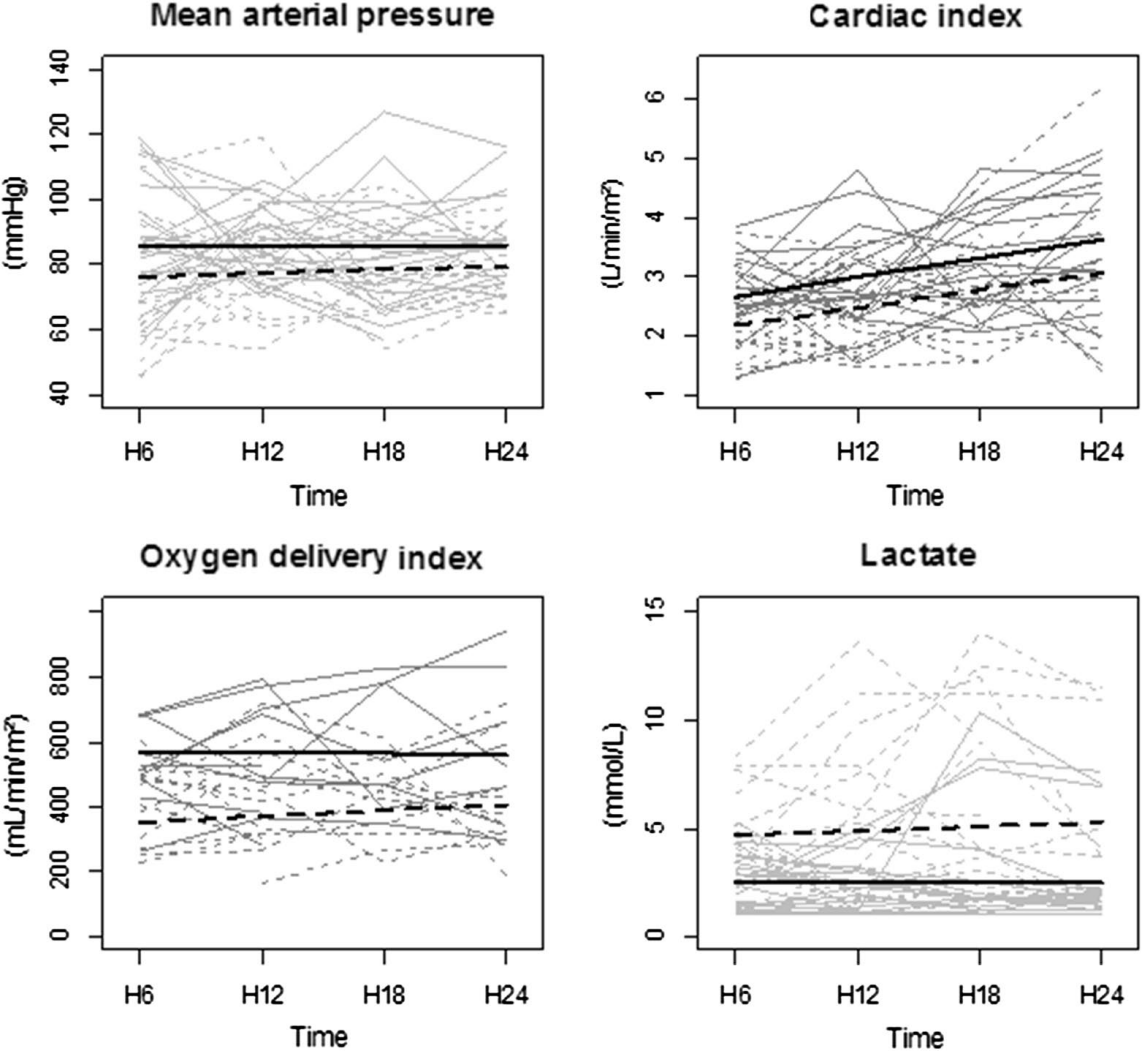
Linear mixed effect models fitted with time of measurement and survival status as fixed effect and random intercept and random slope to account for patient-specific effect. No interaction was found between time and outcome, showing no evidence of differential evolution during the first 24 h. The only parameter that changed significantly with time was cardiac index with a mean increase of 0.05 L/min/m^2^/h. *Continuous lines* indicate survivors, and *discontinuous lines* indicate non-survivors

Each of these parameters was significantly different between day-90 survivor and day-90 deceased patients. No interaction was found between time and outcome regarding macrocirculatory parameters and lactate level. Trends in hemodynamic parameters and serum lactate changes were not different between survivors and non-survivors during the first 24 h. In other words, hemodynamic parameters improved during the first 24 h resuscitation in all patients, but patients who died had lower SV and CI on admission and higher lactate level which remained through the first 24 h (Fig. 3). In this line, we assessed whether the main hemodynamic targets (MAP ≥65 mmHg, UO ≥0.5 mL/kg/h, CI ≥2.5 L/min/m^2^) were reached at the sixth hour after admission. The percentage of patients in whom the therapeutic targets were achieved was significantly lower in non-survivors than in survivors (Table 2). At the 6th h, lactate clearance (%) was not significantly lower in patients who died versus patients who survived [13 (−42 to 39) vs 28 (4–37) %; (*p* = 0.41)].

Despite lower CI and SV in non-survivors, there was no statistical difference between the median pre-hospital and 24-h administered fluid volume in survivors and non-survivors. The time delay between the burn and ICBU admission was also similar between survivors and non-survivors (Table 1).

### Secondary outcomes

In the study population, the 28-day mortality rate was 26 %. Fourteen patients (35 %) developed early MODS in the first 4 days. Sixteen patients (40 %) developed AKI including eight (20 %) who required renal replacement therapy.

Initial in-hospital SV, MAP, SAP, DAP and DO_2_I were significantly lower in patients who developed early MODS [respectively, 23 (21–26) vs 30 (24–41) mL; (*p* = 0.03), 65 (59–83) vs 86 (75–112) mmHg; (*p* = 0.001), 106 (94–116) vs 124 (108–156) mmHg; (*p* = 0.006), 65 (47–75) vs 79 (60–89) mmHg; (*p* = 0.05) and 474 (318–498) vs 561 (484–689) mL/min/m^2^; (*p* = 0.02)]. Furthermore, admission serum lactate levels were significantly higher in these patients 3.9 (2.8–4.8) versus 2.9 (1.5–3.7) mmol/L; (*p* = 0.001).

## Discussion

The main findings of this study are that initial in-hospital SV, CI and DO2I were strongly associated with outcome in critically ill burn patients. SV on admission was the most sensitive and specific predictor of mortality and was independently associated with outcome. Trends in CI and SV were not different between survivors and non-survivors during the first 24 h. Hemodynamic parameters improved during the first 24-h resuscitation in all patients, but patients who died had lower SV and CI on admission, while initial fluid volume (in mL/kg/% TBSA) was not different, suggesting that fluid requirements vary between patients regardless of TBSA.

The main early hemodynamic consequence of severe burns is severe hypovolemia. This condition is mainly related to a generalized disturbance of the endothelial barrier resulting in diffuse capillary leak [16]. Initial hemodynamic profile associates low cardiac output and high systemic vascular resistance [2]. Rapid and adequate intravascular fluid replacement therapy is the cornerstone of the prevention of burn hypovolemic shock. However, optimal hemodynamic targets remain largely unknown and association between hemodynamic parameters, the timing of resuscitation and mortality has been poorly explored.

Several small-sample-size studies have previously identified an association between systemic hemodynamics and outcomes in critically ill burn patients, but none has explored the very early phase [17–23]. Regarding hemodynamics on admission, our results are in agreement with the findings of most of the previous studies and the current pathophysiologic understanding of burn injury shock [2, 17, 18, 20, 21]. In a study including forty-two burn patients with a TBSA >20 % or inhalation injury, Lorente et al. [17] found that a low systemic blood flow and high resistance state was associated with poor outcomes. CI and DO_2_I over the first hours were significantly lower in non-survivors than in survivors. Furthermore, DO_2_I (6 h) was independently associated with mortality. Nevertheless, the mean delay from burn trauma was almost 8 h (vs 3 h in our study) and the delay of Swan Ganz catheters insertion after admission was not mentioned. In our study, only patients with a cardiac output monitoring within the first hour after admission were included. This point raises the question of very early hemodynamic measurements and resuscitation. In the study of Holm et al. [21], 21 patients with an ABSI score ≥6 were included. CI and DO_2_I were significantly lower in non-survivors than in survivors all over the first 72 h after admission. Furthermore, in the Holm et al. study, no multivariate analysis was performed regarding hemodynamic parameters to identify those independently associated with outcome. In older studies using pulmonary artery catheter monitoring [18, 20], low mean CI values throughout the first 72 h were associated with mortality. In these studies, hemodynamic parameters were reported as means over a period of 72 h. As a result, no conclusion regarding the association of the very early hemodynamics and outcome can be made. In the work of Miller et al. [19], only hemoglobin and TBSA showed a statistically significant difference between patients who survived and those who died, but not systemic hemodynamic parameters. However, high CI and low vascular resistances profile suggested that these patients had already been resuscitated. Furthermore, in the study of Branski et al. [24] including pediatric burn patients with a TBSA >40 %, MAP and CI on day 0 were not significantly different between survivors and non-survivors. Nevertheless, the study was not designed to compare hemodynamic parameters on admission, and the hyperkinetic profile on day 0 (mean CI >4 L/min/m^2^) suggested that these patients had already been resuscitated. Furthermore, outcome between pediatric and adult patients is different. Finally, none of the aforesaid studies have tested SV on admission as a predictor of bad outcome in critically ill burn patients. The main results of the aforesaid works are summarized in the Additional file 3: Table S3.

In this study, SV had not better predictive value than CI (ROC AUCs of SV and CI were not significantly different). However, SV might be more directly related to venous return without the influence of HR. Hypovolemic shock might show initial high HR and CI in the normal range despite hypovolemia. In this line, SV may more reflect low intravascular volume compared to CI. In addition, significantly higher serum lactate levels in non-survivors than in survivors persisted throughout the first 24 h. Not surprisingly, our findings confirm previous data on serum lactate level as early predictor of outcome in burn patients [23, 25, 26]. Worse outcomes associated with high serum lactate levels are mainly explained by hypoperfusion induced by burn shock and the stress reaction related to the burn injury severity.

Delayed or inadequate fluid replacement therapy results in shock, tissue hypoperfusion and sustained MODS, some being irreversible and leading to death [13]. However, the “ideal” intravascular fluid volume replacement strategy remains controversial [27, 28]. In most burn centers, fluid therapy in the first 24 h post-injury is administrated on the basis of the empiric Parkland formula and the adequacy of tissue perfusion in critically ill burn patients is mainly evaluated on arterial blood pressure and UO [29]. Another approach is the goal-directed fluid resuscitation therapy based on physiological monitoring and hemodynamic targets [30–33].

In a recent systematic review and meta-analysis, a decrease in mortality was seen with the use of hemodynamic alternative endpoints rather than hourly UO (risk ratio = 0.77; 95 % confidence interval [0.42–0.85]; *p* < 0.004) [5]. In our study, the percentage of patients in whom the main hemodynamic targets (CI, MAP and UO) were achieved in the 6th h was significantly lower in non-survivors than in survivors. No interaction was found between time and outcome, suggesting that non-survivors presented with altered hemodynamic parameters due to initial under-resuscitation. In other words, resuscitation was able to improve CI in all patients, but non-survivors had lower initial values that remained lower than for survivors for the first 24 h.

It is worth mentioning potential limitations in this study. First, this study was retrospective. However, parameters were prospectively collected in a standardized protocol. Thus, it is unlikely that this potential limitation affected our results. Second, we reported hemodynamic variables as an initial single measurement. In a shock state, potential rapid changes in hemodynamics can occur, and a more appropriate approach could be to take into account both the duration and severity of hypotension and hypoperfusion. Finally, the small sample size of patients limited the number of variables entered in the multivariate analysis to three (SV, SAPS II and TBSA). Nonetheless, a physiological interdependence exists between many hemodynamic and oxygenation parameters and as such, examining other associations would have added little. Finally, the observational design prevents to draw any firm conclusion regarding the causal relationship between the resuscitation strategy and outcome.

Notwithstanding, this study has several strengths. First, to the best of our knowledge, this is one of the largest recent (<20 years) studies assessing the association between hemodynamic status and outcome in adult critically ill burn patients, especially in the very early period. Second, in this work, the mean delay from burn trauma was almost 3 h (vs 7–8 h in the previous studies) [17, 18] and only patients with a cardiac output monitoring within the first hour after admission were included. This allowed the collection of truly early hemodynamic measurements. Finally, we identified initial SV and CI as strongly associated with outcome, suggesting early under-resuscitation in non-survivors. Integrating hemodynamic parameters, timing and fluid volume suggests that fluid requirement varies between patients, and very early hemodynamic monitoring may serve at tailoring fluid resuscitation in critically ill burn patients.

## Conclusion

In this study, SV and CI on admission in the very early period were independently associated with 90-day mortality regardless of TBSA. Very early SV and CI monitoring may help in detecting under-resuscitated patients. Future prospective interventional studies should explore the impact of early GDT in these specific patients.

## Additional files

10.1186/s13613-016-0192-y Screening flowchart of the study population. *TBSA* total body surface area burn-injured, *ICBU* intensive care burn unit.
10.1186/s13613-016-0192-y The survival curve from admission to day 90.
10.1186/s13613-016-0192-y Characteristics of the main clinical studies assessing the association between hemodynamics on admission and outcome in critically ill burn patients.

## Abbreviations

GDT: goal-directed therapy
ICBU: intensive care burn unit
TBSA burn: total body surface area burn
CVC: central venous catheter
SVC: superior vena cava
MODS: multiple organ dysfunction syndrome
SOFA score: Sequential Organ Failure Assessment score
BMI: body mass index
SAPS II: Simplified Acute Physiology Score II
ABSI: Abbreviated Burn Severity Index
AKI: acute kidney injury
HR: heart rate
CI: cardiac index
SV: stroke volume
SAP: invasive systolic arterial pressure
MAP: invasive mean arterial pressure
DAP: invasive diastolic arterial pressure
SVRI: systemic vascular resistance index
CVP: central venous pressure
UO: urine output
ScvO_2_: central venous oxygen saturation
DO_2_I: oxygen delivery index
PCO_2_ gap: central venous–arterial PCO_2_ difference
TPTD: transpulmonary thermodilution
IQR: interquartile range
ROC: receiver operating characteristic curve
AUC: area under the curve
ANOVA: analysis of variance repeated measures
