# Supplementary figures and images for: Low cardiac index and stroke volume on admission are associated with poor outcome in critically ill burn patients: a retrospective cohort study
Source: Ann Intensive Care. 2016 Sep 13;6(1):87. doi: 10.1186/s13613-016-0192-y (PMC5020003; doi:10.1186/s13613-016-0192-y)

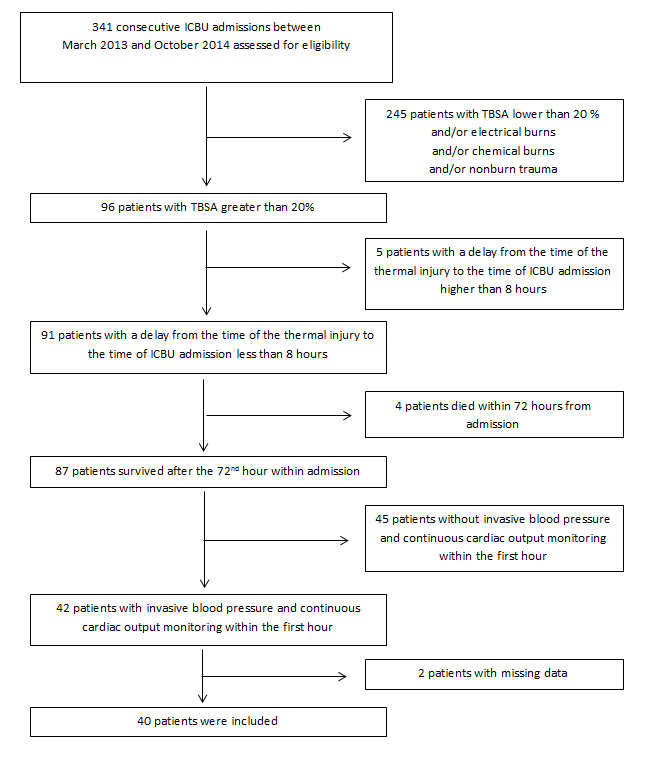

Supplement: Supplementary file 1 — 10.1186/s13613-016-0192-y Screening flowchart of the study population. TBSA total body surface area burn-injured, ICBU intensive care burn unit. [file 13613_2016_192_MOESM1_ESM.tif]

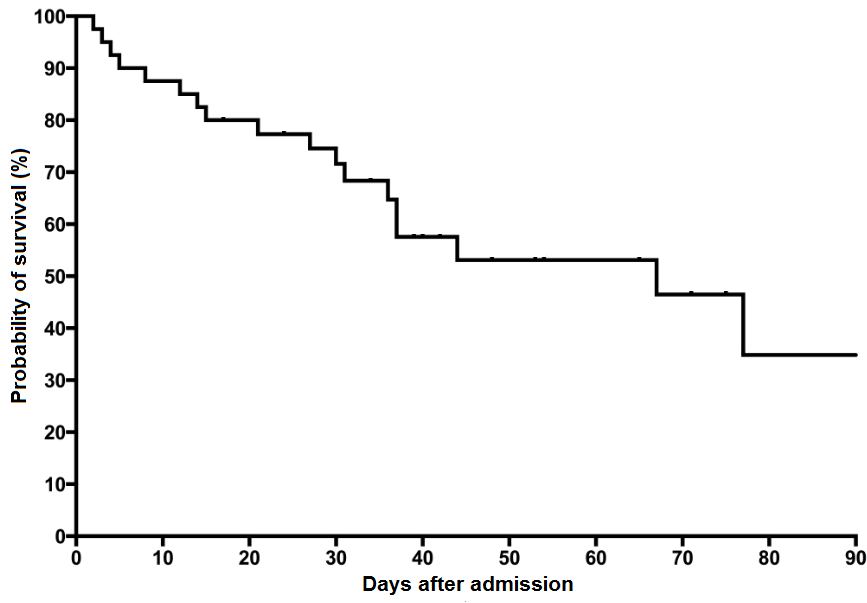

Supplement: Supplementary file 2 — 10.1186/s13613-016-0192-y The survival curve from admission to day 90. [file 13613_2016_192_MOESM2_ESM.tif]
