# Supplementary material for: Low cardiac index and stroke volume on admission are associated with poor outcome in critically ill burn patients: a retrospective cohort study
Source: Ann Intensive Care. 2016 Sep 13;6(1):87. doi: 10.1186/s13613-016-0192-y (PMC5020003; doi:10.1186/s13613-016-0192-y)
Supplement: Supplementary file 3 — 10.1186/s13613-016-0192-y Characteristics of the main clinical studies assessing the association between hemodynamics on admission and outcome in critically ill burn patients. [file 13613_2016_192_MOESM3_ESM.pdf]

Table S1. Characteristics of the main clinical studies assessing the association between hemodynamics on admission and outcome in severely burn patients.

Study (year)	Design	Study population	Population size	TBSA (%) and severity scores	Delay from burn trauma	Hemodynamic monitoring	Key endpoints and main results
Lorente, 2000 [17]	Retrospective cohort	Adult patients	42	-TBSA:<br>S : 36 ± 14 vs<br>NS : 45 ± 22	S : 7 ± 2 hrs<br>vs<br>NS : 8 ± 2 hrs	PAC and gastric tonometer	- In-hospital mortality (38%).<br>- DO_2I (6hrs) was independently associated with mortality.<br>- CI over the first 12 hrs and DO_2 over the first 42 hrs were significantly lower in NS than in S.<br>- Patients with a P [i-a] CO_2 difference (6 hrs) ≥ 10 torr had a worse outcome
Holm, 2000 [21]	Prospective observational	Adult patients	21	- TBSA : 40 (20-67)<br>- ABSI: 9 (6-11)	NR	TPTD technique	- In-hospital mortality (29 %)<br>- CI and DO_2 over the first 75 hrs were significantly lower in patients who died
Bernard, 1994 [18]	Retrospective cohort	Adult patients	38	- TBSA : 64 ± 15<br>- UBS: 241 ± 67	7 (1 – 36) hrs	PAC	- In-hospital mortality (50 %)<br>- Low mean CI and DO_2I over a period of 72 hrs were associated with mortality
Shiller, 1995 [20]	Retrospective cohort	Adult patients	53	- TBSA : 40	NR	PAC	- In-hospital mortality (30 %)<br>- Low mean CI and DO_2I over a period of 72 hrs were associated with bad outcome
Miller, 1994 [19]	Retrospective cohort	Adult patients	22	- TBSA :<br>S : 19 (± 20) vs<br>NS : 43 (± 7)<br>- APACHE II :<br>S : 11 (± 5) vs<br>NS : 16	NR	PAC	- In-hospital mortality (77%)<br>- Hemodynamic parameters showed no significant difference between S and NS<br>- Hb and TBSA were associated with worse outcome
Branski, 2011 [24]	Prospective observational	Pediatric patients	79	- TBSA : 64 (± 35)	NR	TPTD technique	- In-hospital mortality (20 %)<br>- MAP and CI were not significantly different between S and NS<br>- EVLW was significantly higher in NS after the sixth hour after admission

TBSA burn Total body surface area burn, *S* Survivors, *NS* Non-survivors, *PAC* Pulmonary artery catheter, *P [i-a]* CO_2 Difference between arterial and gastric mucosal PCO_2 , *ABSI* Abbreviated Burn Severity Index, *TPTD* Transpulmonary thermodilution, *CI* Cardiac index, DO_2I Oxygen delivery index, *NR* Not reported, *UBS* Unit Burn Standard score , *APACHE II Acute Physiology And Chronic Health Evaluation II score*, *EVLW* Extravascular lung water. Data are expressed in mean (± SD).
